# Point-of-care lung ultrasound - a rapid and reliable diagnostic tool for emergency physicians treating patients with acute dyspnea in high-volume emergency departments

**DOI:** 10.1007/s10140-025-02343-4

**Published:** 2025-04-22

**Authors:** Irina Ciumanghel, Eliza Barbuta, Adi-Ionut Ciumanghel, Iulian Buzincu, Gabriela Grigorasi, Diana Cimpoesu

**Affiliations:** 1 University of Medicine and Pharmacy “Gr. T. Popa” Iasi, Iași, Romania; 2 Emergency Department, Clinical Emergency County Hospital “St. Spiridon” Iasi, Iași, Romania; 3 Anesthesiology and Intensive Care Department, Clinical Emergency County Hospital “St. Spiridon” Iasi, Iași, Romania

**Keywords:** Lung ultrasound, Point-of-care lung ultrasound, Emergency physician, Acute dyspnea

## Abstract

**Purpose:**

Acute dyspnea is a common presenting symptom in the Emergency Department (ED). The study aims to assess the concordance between emergency physician diagnosis (i.e., initial rapid assessment at ED admission including point-of-care lung ultrasound - PoC-LUS) and attending physician diagnosis (i.e., hospital admission diagnosis which also includes CT scans) in patients presenting with dyspnea.

**Method:**

We performed a prospective pilot observational study in the ED of tertiary care university hospital between 31.01.2022 and 03.09.2024. We included dyspneic patients presented when the physician involved in the study was on call.

**Results:**

A total of 103 patients were included (mean age, 70±16.1 years). An excellent agreement was found between emergency physician and attending physician diagnosis for all etiologies of dyspnea: pleural effusion (Cohen’s kappa coefficient 1 for bilateral, 0.844 for right, 0.790 for left pleural effusion), pneumonia (κ = 0.979 for right, κ = 0.930 for left pneumonia), bronchopneumonia (κ = 0.912), acute pulmonary edema (κ = 1), chronic obstructive pulmonary disease exacerbation (κ = 0.904), pleuropulmonary tumors (k = 0.884), acute respiratory distress syndrome – ARDS (κ = 1), (*p* < 0.001 for all). The median(±SD) time needed to complete the emergency physician diagnosis was 16(±4) minutes and the median(±SD) time needed to complete the attending physician diagnosis was 480(±112) minutes.

**Conclusion:**

In patients presenting in the ED with dyspnea, PoC-LUS guided emergency physician diagnosis has a very good diagnosis performance. The time needed to complete the emergency physician diagnosis is much lower than the time needed to complete the attending physician diagnosis. Given its availability, PoC-LUS is a useful tool for the assessment of patients presenting with dyspnea.

**Graphical Abstract:**

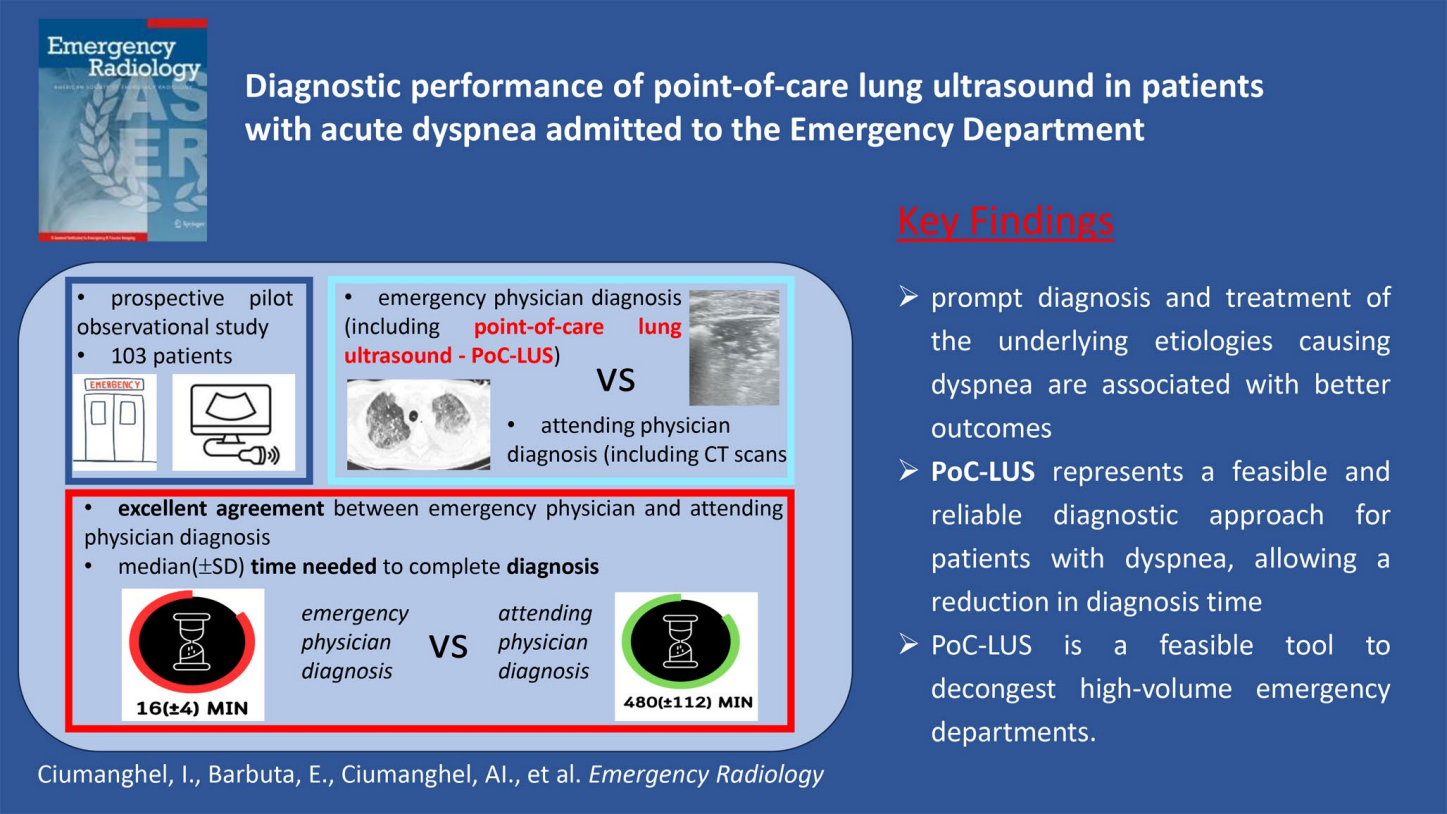

## Background

Acute dyspnea is a common presenting symptom in the Emergency Department (ED) [1] and it can be caused by many pulmonary and cardiac diseases (pleural effusion, pneumonia, bronchopneumonia, acute pulmonary edema, chronic obstructive pulmonary disease exacerbation, pleuropulmonary tumors, acute respiratory distress syndrome - ARDS) [2]. A rapid and accurate etiological diagnosis can guide an early and appropriate treatment and may be associated with better outcome, especially in critically ill patients [3, 4].

An anamnestic and clinical examination can direct a diagnosis suspicion regarding the etiology of the acute dyspnea, but this needs to be corroborated by laboratory and imagistic testing, the results of these being often delayed [5, 6].

Point-of-care lung ultrasonography (PoC-LUS) may reduce the time to a complete diagnosis and the use of other radiation-based imaging techniques like chest x-ray or computed tomography (CT) in the ED, but this approach needs to be validated [7, 8].

This study started from the clinical reality that our ED is a very overcrowded one and our radiology department is overwhelmed by the number of X-rays and CT scans. Therefore, the radiologists are overworked, the equipment frequently breaks and most important, waiting times are increased. Although CT scan is the gold standard for lung imaging, PoC-LUS can identify the cause of acute dyspnea, can improve the patients flow in the ED and can prevent radiant and expensive imagistic tests.

In this study we assessed the concordance between *emergency physician diagnosis* (i.e., initial rapid assessment at ED admission including medical history, clinical evaluation, ABGs and PoC-LUS) and *attending physician diagnosis* (i.e., hospital admission diagnosis which also includes laboratory hematological and biochemical tests, CT scans and other specialist assessments as needed) in patients with dyspnea presenting in the ED.

## Methods

### Study design

We conducted a prospective pilot observational study in the ED of the Clinical Emergency County Hospital “St. Spiridon”, Iasi, Romania from 31.01.2022 to 03.09.2024. This hospital is the largest in the North-East region of Romania with a total of 1153 beds, is an academic medical center and the ED has an annual of 83.000 presentations.

The study protocol was approved by the ethics committee of the hospital (nr. 73/2024) and respected the statements of the declaration of Helsinki, with informed consent from patients or their legal representatives being obtained prior to the recruitment for all the patients included in the study.

The primary objective of our study was to assess the concordance between *emergency physician diagnosis* and *attending physician diagnosis* in patients with dyspnea presenting in the ED, considering the *attending physician diagnosis* (i.e., hospital admission diagnosis) as the correct one. The *emergency physician diagnosis* was done by the emergency physician responsible for the study and was formulated in the shortest possible time from the presentation, based on medical history, clinical evaluation, ABGs analysis and PoC-LUS, without having access to the laboratory hematological and biochemical tests, CT scans and other specialist assessments. This *emergency physician diagnosis* was compared with the *attending physician diagnosis* - hospital admission diagnosis formulated by another clinician who admitted the patient on ward or in intensive care (cardiologist, internist, pneumologist, thoracic surgeon) who had access to all the patient data (including also medical history, clinical evaluation, ABGs analysis, laboratory hematological and biochemical tests, CT scans and other specialist assessments as needed) but was not aware of the PoC-LUS results.

The secondary objective of the present study was to compare the time needed to complete the *emergency physician diagnosis* and the time needed to complete the attending physician diagnosis.

### Study population

Inclusion criteria:

- Patients above 18 years of age who presented to the ED with primary complaint of acute dyspnea (either sudden onset or increase in severity of chronic dyspnea) when the emergency physician involved in the study was on call.

Exclusion criteria:

- Consent declined.
- Patient younger than 18.
- Trauma patients.
- Patients with poor acoustic window (obesity, chest deformities, subcutaneous emphysema).

### Study protocol

At the ED presentation, *emergency physician diagnosis* was formulated based on medical history, the physical examination, a 12-lead electrocardiography (ECG) and ABGs analysis, carried out by the attending team (study team). Based on this assessment a preliminary diagnosis was formulated. After this, PoC-LUS was performed by the principal study investigator, an emergency medicine (EM) consultant with a Lung Ultrasound (LUS) diploma, with 4 years of LUS experience.

### Medical history and clinical evaluation

Patients’ medical history, vital signs (systolic, diastolic, and mean arterial pressure, heart rate, respiratory rate, peripheral oxygen saturation – SpO_2_) and clinical examination were recorded by the enrolling physician.

We also recorded the intubated and mechanically ventilated patients before being admitted in the ED or who needed oxygen therapy or mechanical ventilation (invasive or non-invasive) during the ED stay. For all these patients we recorded the inspiratory oxygen concentration (FiO_2_), and we calculated peripheral arterial oxygen saturation to the inspired fraction of oxygen (SpO_2_/FiO_2_). Also, we recorded the patients on vasopressor or inotrope medication in the ED and we calculated for all the patients admitted in the study the shock index (heart rate divided by systolic blood pressure).

### Lung ultrasonography

For PoC-LUS we used a Vivid T8 GE ultrasound machine. The protocol we used consisted in dividing each hemithorax in 6 quadrants: superior and inferior parts of the anterior, lateral and posterior chest wall.

LUS was performed using any of the two transducers: linear (4.0–13.0 MHz) and convex (1.3-4.0 MHz). We started with the convex probe, ideal for the lateral quadrants’ examinations or in case of patients with a high BMI. When we wanted to better visualize the superficial layers such as the pleura and when scanning the anterior zone we used the linear transducer. In case of using the convex transducer as a preset we used *lung* or *abdomen*, and in case of the linear transducer we used *lung* or *small parts*. The probe was oriented both transversely (intercostally) and longitudinally.

The scanning was performed with the patient in supine or sitting position for the anterior and lateral examination of the thorax and in sitting position for the posterior examination. In order to examine the posterior thorax of intubated patients we mobilized them laterally on both sides.

### Major pathologies identified

**Fig. 1:**
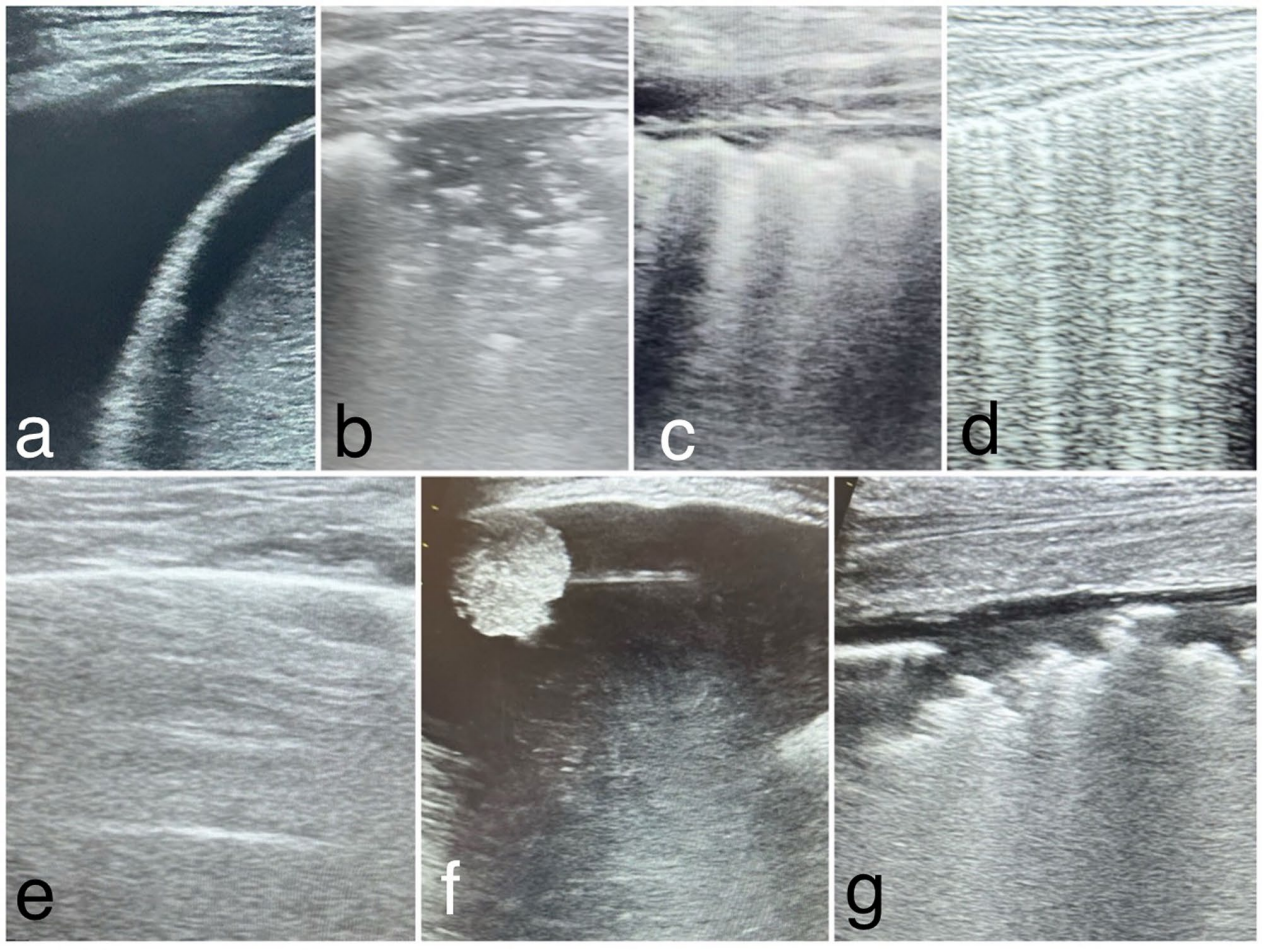
Images of LUS findings in major pleuro-pulmonary pathologies. **a**) Pleural effusion; **b**) Pneumonia; **c**) Bronchopneumonia; **d**) Acute pulmonary edema; **e**) COPD exacerbation; **f**) Pleuropulmonary tumors; **g**) ARDS. (images from personal archive)

PoC-LUS is an important tool in the diagnosis and management of major pathologies like pleural effusion, pneumonia, bronchopneumonia, acute pulmonary edema, COPD (chronic obstructive pulmonary disease) exacerbation, pleuropulmonary tumors and ARDS (Table 1; Fig. 1).

**Table 1:** PoC-LUS findings in major pleuro-pulmonary pathologies

Pathologies	LUS findings
Pleural effusion	An anechoic area between the parietal and visceral pleura
Pneumonia	Pathological B lines, fragmented pleural line, dinamic air bronchograms, lung “hepatization”
Bronchopneumonia	Pathological B lines, fragmented pleural line bilaterally, bilateral consolidations of various size, lung sliding might be absent
Acute pulmonary edema	Multiple B lines bilaterally on all zones, normal pleural line, present lung sliding
COPD exacerbation	A line profile (with abnormal ABGs)
Pleuropulmonary tumors	Well delimited round/oval consolidations, adherent to the parietal or visceral pleura
ARDS	Pathological B lines distributed uneven bilaterally, consolidations with air bronchograms bilaterally, very fragmentated pleural line, absent lung sliding

COPD = chronic obstructive pulmonary disease; ARDS = acute respiratory distress syndrome

### Laboratory hematological and biochemical tests

For all the patients enrolled in the study we recorded the laboratory data: hematological tests (white blood, neutrophil, lymphocyte and platelet count), standard coagulation tests (fibrinogen, prothrombin time, activated partial thromboplastin time, INR), biochemical laboratory tests (C reactive protein, procalcitonin, ferritin, sodium, potassium, chloride, serum bicarbonate, glycemia, urea, creatinine, ALT, AST, bilirubin, direct bilirubin, CK, CK-MB, LDH, D-dimers, NT-proBNP). For all patients we measured the arterial blood gases: pH, PCO_2_, PaO_2_, BE and lactate. We calculated the ratio of arterial oxygen partial pressure to fractional inspired oxygen (PaO_2_/FiO_2_) and neutrophil-to-lymphocyte ratio (NLR).

### CT scans

**Fig. 2:**
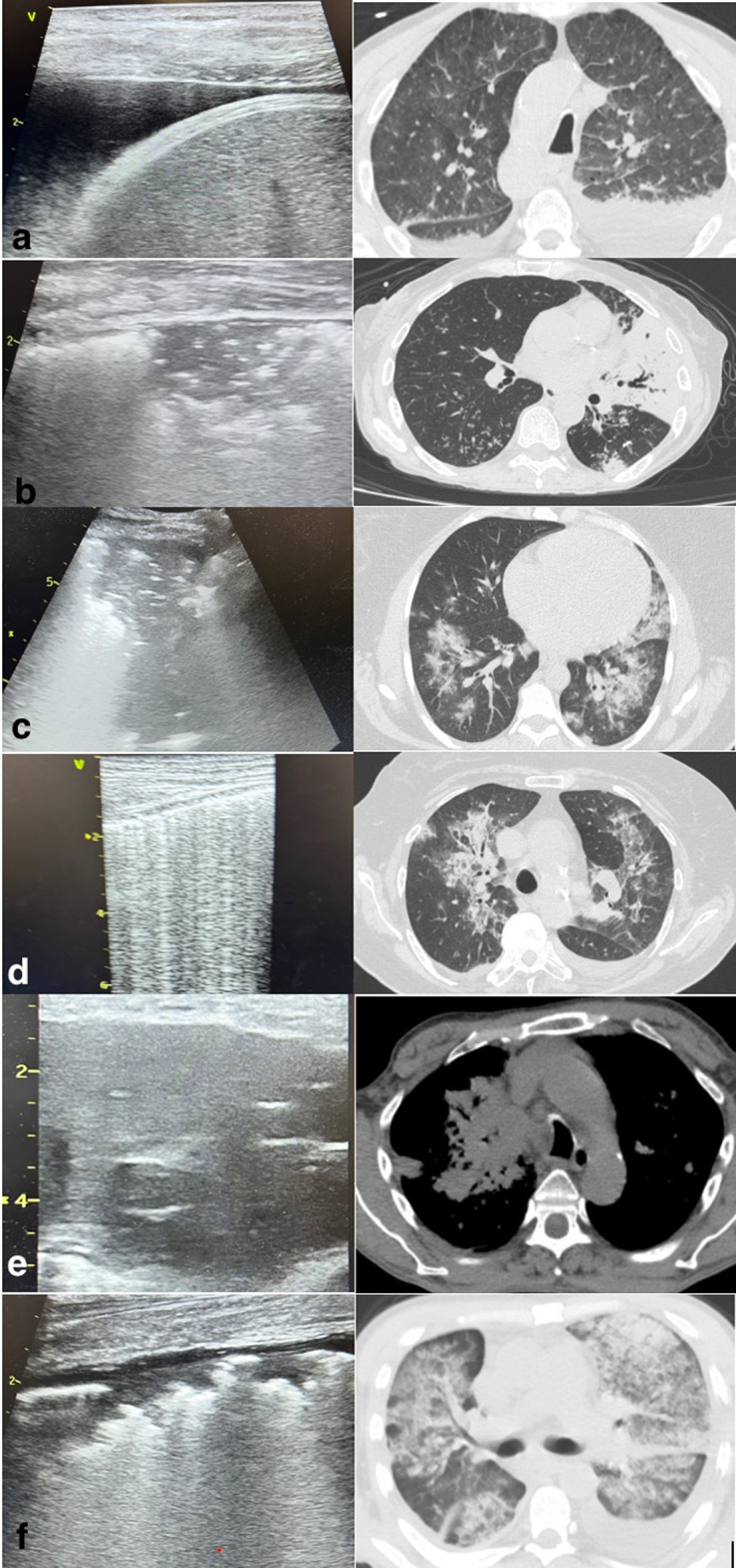
Images of LUS findings compared with thoracic CT scans. **a**) Pleural effusion; **b**) Pneumonia; **c**) Bronchopneumonia; **d**) Acute pulmonary edema; **e**) Pleuropulmonary tumors; **f**) ARDS. (images from personal archive)

Blinded to the PoC-LUS results, chest CT scans were assessed by experienced radiologists (according to the standardized protocol) who had access to the clinical and laboratory data. Chest CT was performed using a 128 slices CT (Philips CT Scanner) with the patient in supine position. A comparison between the images of LUS and CT scans can be seen in Fig. 2.

### Statistical analysis

Statistical analysis was performed using the Jamovi ver. 2.4 (2023). The sensitivity, specificity, positive predictive value (PPV), and negative predictive value (NPV) were calculated for each diagnostic category. To measure agreement between the rapid initial diagnosis and the hospital admission diagnosis we used The Cohen’s Kappa for 2 Raters (κ). Statistical significance was assessed using p-values calculated for each k statistic. A p-value of < 0.05 was considered statistically significant.

## Results

### Descriptive analysis - demographic data and baseline information about the study population

One hundred and three patients were included in the study, the mean (±SD) age was 70±16.1 years, 56.3% were of male gender and 83.5% of the patients’ presented comorbidities, the main being cardiac (65.04%), vascular (31.06%), neurological (27.1%), metabolic (21.4%) and chronic respiratory (18.4%).

During the ED stay, 54 patients (52.4%) received supplementary oxygen, 13 patients (12.6%) needed non-invasive mechanical ventilation and 26 (25,2%) needed invasive mechanical ventilated. The mean (±SD) admission SpO_2_, SpO_2_/FiO_2_ and PaO_2_/FiO_2_ were 90.6±8.9, 313.4±136 and 261.8±124.

Twenty-seven patients received vasopressors and the admission mean (±SD) shock index was 0.86±0.33.

The baseline demographic, clinical and laboratory data of the patients are presented in Tables 2 and 3.

**Table 2:** Patient characteristics and clinical parameters

Parameter	Value
Age (yrs ± SD)	70 ± 16.1
Male Gender, *n* (%)	58 (56.3%)
Comorbidities, *n* (%)	
Cardiovascular	67 (65.04%)
Neurological	28 (27.1%)
Vascular	32 (31.06%)
Respiratory	19 (18.4%)
Metabolic	22 (21.4%)
Renal	16 (15.5%)
Gastrointestinal	16 (15.5%)
Neoplastic	6 (5.8%)
Respiratory rate (breaths/min), median [IQR]	18 [8.5]
SpO₂ (%), mean ± SD	90.6 ± 8.9
PaO₂ (mmHg), mean ± SD	90.9 ± 67.9
SpO₂/FiO₂ Ratio, mean ± SD	313 ± 136
PaO₂/FiO₂ Ratio, mean ± SD	261 ± 124
Oxygen requirement, *n* (%)	54 (52.4%)
Non-invasive Mechanical Ventilation, *n* (%)	13 (12.6%)
Invasive Mechanical Ventilation, *n* (%)	26 (25.2%)
Systolic Blood Pressure (mmHg), mean ± SD	131 ± 31.8
Diastolic Blood Pressure (mmHg), mean ± SD	78 ± 17.6
Mean Arterial Pressure (mmHg), mean ± SD	96.7 ± 21.2
Heart Rate (beats/min), median [IQR]	100 [35]
Shock Index, mean ± SD	0.86 ± 0.33
Need for Vasopressor Support, *n* (%)	27 (26.2%)

SD = standard deviation; IQR = interquartile range; SpO₂ = peripheral arterial oxygen saturation; PaO₂ = arterial oxygen partial pressure; SpO₂/FiO₂ = ratio peripheral arterial oxygen saturation to the inspired fraction of oxygen; PaO₂/FiO₂ = ratio of arterial oxygen partial pressure to fractional inspired oxygen

**Table 3:** Laboratory parameters

Parameter	Value
WBC (×10^3^/L), median [IQR]	11.8 [10.9]
Neutrophil Count (×10^3^/L), median [IQR]	9.28 [8.38]
Lymphocyte Count (×10^3^/L), median [IQR]	1.23 [1.15]
NLR, median [IQR]	7.04 [10.2]
PLT (×10^3^/L), median [IQR]	226 [109]
Fibrinogen (mg/dL), mean ± SD	418.5 ± 139
PT (seconds), median [IQR]	14.3 [3.4]
INR, median [IQR]	1.29 [0.32]
APTT (seconds), median [IQR]	29 [8.2]
CRP (mg/dL), median [IQR]	6.03 [17.4]
Ferritin (ng/mL), median [IQR]	252 [395]
Sodium (mmol/L), median [IQR]	138 [7]
Potassium (mmol/L), median [IQR]	4.5 [1]
Chloride (mmol/L), median [IQR]	103 [8.5]
Serum Bicarbonate (mmol/L), median [IQR]	21.5 [5.45]
Glycemia (mg/dL), median [IQR]	131 [85.5]
Urea (mg/dL), median [IQR]	55 [52.5]
Creatinine (mg/dL), median [IQR]	1.15 [0.96]
ALT (U/L), median [IQR]	23 [35.8]
AST (U/L), median [IQR]	34 [28.8]
Total Bilirubin (mg/dL), median [IQR]	0.88 [1.1]
Direct Bilirubin (mg/dL), median [IQR]	0.43 [0.44]
CK (U/L), median [IQR]	83 [113]
CK-MB (ng/mL), median [IQR]	25 [23.3]
LDH (U/L), median [IQR]	264 [118]
pH, median [IQR]	7.38 [0.2]
BE (mmol/L), median [IQR]	-2.7 [9.4]
Lactate (mmol/L), median [IQR]	1.8 [2.5]
PCT (ng/mL), median [IQR]	0.41 [1.11]
D-dimers (mg/L), median [IQR]	2.75 [3.25]
NT-proBNP (pg/mL), median [IQR]	2443 [9148]

SD = standard deviation; IQR = interquartile range; WBC = white blood cell count; NLR = neutrophil-to-lymphocyte ratio; PLT = platelet count; PT = prothrombin time; INR = International Normalized Ratio; APTT = Activated Partial Thromboplastin Time; CRP = C-Reactive Protein; ALT = alanine aminotransferase; AST = aspartate aminotransferase; CK = creatine kinase; CK-MB = creatine kinase-MB; LDH = lactate dehydrogenase; BE = base excess; PCT = procalcitonin; NT-proBNP = N-terminal pro-BNP

### Primary outcome

#### Comparation between emergency physician diagnosis (including PoC-LUS) and attending physician diagnosis (including thoracic CT scan) for pleural effusion

Overall, 44 patients (42.7%) had bilateral pleural effusion, 17 patients (16.5%) right pleural effusion and 5 patients (4.9%) left pleural effusion.

Comparing to the hospital admission diagnosis, the sensitivity and specificity of initial rapid assessment was 100% and 100% for bilateral pleural effusion, 76.5% and 100% for right pleural effusion, 80% and 99% for left pleural effusion.

The concordance between initial rapid assessment and hospital admission diagnosis was analyzed using Cohen’s kappa test. There was a strong agreement between initial and admission diagnosis: Cohen’s kappa was 1.00, 0.844 and 0.790 for bilateral pleural effusion, right pleural effusion and left pleural effusion, respectively. The level of agreement was statistically significant for all the three diagnoses (*p* < 0.001) (Table 4).

**Table 4:** Comparation between emergency physician diagnosis and attending physician diagnosis

Cause of dyspnea	No. of patients	Prevalence	Sensitivity	Specificity	Positive predictive value	Negative predictive value	Cohen’s Kappa	*p*-value
Bilateral pleural effusion	44	42.7%	100.0%	100.0%	100.0%	100.0%	1.00	< 0.001
Right pleural effusion	17	16.5%	76.5%	100.0%	100.0%	95.6%	0.844	< 0.001
Left pleural effusion	5	4.9%	80.0%	99.0%	80.0%	99.0%	0.790	< 0.001
Right pneumonia	37	35.9%	100.0%	98.5%	97.4%	100.0%	0.979	< 0.001
Left pneumonia	31	30.1%	93.3%	98.6%	96.7%	97.3%	0.930	< 0.001
Bronchopneumonia	14	13.6%	85.7%	100.0%	100.0%	97.8%	0.912	< 0.001
Acute pulmonary oedema	10	9.7%	100.0%	100.0%	100.0%	100.0%	1.00	< 0.001
COPD in exacerbation	6	5.8%	83.3%	100.0%	100.0%	99.0%	0.904	< 0.001
Pleuro-pulmonary tumors	5	4.9%	80.0%	100.0%	100.0%	99.0%	0.884	< 0.001
ARDS	2	1.9%	100.0%	100.0%	100.0%	100.0%	1.00	< 0.001

COPD = chronic obstructive pulmonary disease; ARDS = acute respiratory distress syndrome

#### Comparation between emergency physician diagnosis (including PoC-LUS) and attending physician diagnosis (including thoracic CT scan) for pneumonia

Thirty-seven patients (*35.9*%) had right pneumonia as a hospital admission diagnosis. The initial rapid assessment had a 100% sensitivity and 98.5% specificity in identifying the right pneumonia. Left pneumonia was found in 31 patients (30.1%) and the sensitivity and specificity for rapid assessment was 93.3 and 98.6, respectively. The correlation between initial rapid assessment and hospital admission diagnosis was strong for both right (κ = 0.979) and left (κ = 0.930) pneumonia (*p* < 0.001 for both) (Table 4).

#### Comparation between emergency physician diagnosis (including PoC-LUS) and attending physician diagnosis (including thoracic CT scan) for Bronchopneumonia

Bronchopneumonia was the admission diagnosis for 14 patients (13.6%). The sensitivity, specificity and Cohen’s kappa coefficient for rapid assessment were 85.7%, 100% and 0.912 (*p* < 0.001), respectively (Table 4).

#### Comparation between emergency physician diagnosis (including PoC-LUS) and attending physician diagnosis (including thoracic CT scan) for acute pulmonary edema

Ten patients have acute pulmonary edema as an admission diagnosis. The accuracy of rapid assessment in identifying patients with acute edema was very good: 100% sensibility, 100% specificity and κ = 1 (*p* = 0.001) (Table 4).

#### Comparation between emergency physician diagnosis (including PoC-LUS) and attending physician diagnosis (including thoracic CT scan) for COPD exacerbation

COPD exacerbation was the cause of dyspnea for 6 patients (5.8%). The initial assessment has an 83.3% sensitivity, 100% specificity for identifying COPD exacerbation as a cause of dyspnea. The correlation Cohen’s Kappa coefficient was 0.904, showing a strong correlation between initial rapid assessment and the admission diagnosis (*p* < 0.001) (Table 4).

#### Comparation between emergency physician diagnosis (including PoC-LUS) and attending physician diagnosis (including thoracic CT scan) for pleuropulmonary tumors

Five patients (4.9%) had dyspnea due to the pleuropulmonary tumors. The initial rapid assessment had an 80% sensitivity, 100% specificity for identifying the pleuropulmonary tumors. The correlation between initial rapid assessment and hospital admission diagnosis was strong for pleuropulmonary tumors (κ = 0.884, *p* < 0.001) (Table 4).

#### Comparation between emergency physician diagnosis (including PoC-LUS) and attending physician diagnosis (including thoracic CT scan) for acute respiratory distress syndrome (ARDS)

Only 2 patients in our study had ARDS. The sensibility, specificity, and Cohen’s kappa coefficient for these two patients were 100%,100%, and 1, respectively (Table 4).

### Secondary outcome

The median(±SD) time needed to complete *emergency physician diagnosis* was 16(±4) minutes and the median(±SD) time needed to complete *attending physician diagnosis* the attending physician was 480(±112) minutes.

## Discussions

The most important finding of our study is that we identified a strong correlation between the *emergency physician diagnosis (*medical history, clinical evaluation, ABGSs and PoC-LUS) and *attending physician diagnosis* (which also includes laboratory hematological and biochemical tests, CT scans, and other specialist assessments as needed) in patients who presented in the ED with acute dyspnea. This is very important for the high-volume EDs where the imagistic laboratory is overwhelmed by the high number of investigations and the time to a complete diagnosis is very high.

While medical history, clinical evaluation, and arterial blood gases are important tools, their diagnostic value is limited in unselected patients presenting with acute dyspnea in the emergency department [9, 10]. The clinical suspicion of the diagnosis must be confirmed through imaging assessment.

Although thoracic CT scan is the gold standard for lung imaging, it is radiating, expensive, and the time to scan and to obtain an interpretation is long [9]. Point of care lung ultrasound (PoC-LUS) is a rapid, immediately available, non-invasive bedside technique that allows for the diagnosis of many respiratory pathologies with good accuracy and greater speed compared to the gold standard lung imaging technique (CT scan) [11]. In our study, the agreement coefficient between *emergency physician diagnosis* (including PoC-LUS) and *attending physician diagnosis* (including thoracic CT scan) was high for all the pathologic entities evaluated: pleural effusion, pneumonia, bronchopneumonia, acute pulmonary edema, COPD exacerbation, pleuropulmonary tumors or ARDS. The same conclusion was reached by Staub and all in their review assessing the diagnostic accuracy of PoC-LUS in the patients presenting in the emergency department with acute respiratory symptoms due to pneumonia, COPD exacerbation or acute pulmonary edema [12].

The mean time needed to complete an accurate diagnosis in the *emergency physician diagnosis* was 16±4 min in comparation with an *attending physician diagnosis* which tacked 480±112 min. Zanobetti et al.l compared the concordance and the time needed for an emergency physician using POC-LUS versus an emergency physician using standard approach (without PoC-LUS) to formulate a correct diagnosis in patients presenting with acute dyspnea in ED [13]. They found a good concordance between these two approaches, but the time needed to formulate the diagnosis with using ultrasound was significantly lower than without PoC-LUS (24±10 min versus 186±72 min). This is potentially very important for an emergency diagnosis in which a rapid treatment may be associated with better prognosis.

The pressure on EDs is getting higher every day, demanding high quality care despite the constantly increase of admissions. Several studies have reported a relationship between overcrowding in the ED and mortality [14, 15]. Improvements in the survival rate in the critically ill patients admitted in the EDs are directly related to early recognition and rapid initiation of treatment. Integrating PoC-LUS in an *emergency physician diagnosis* was associated with a decreased time to diagnosis without impacting the accuracy in our study. However, no data is available regarding the impact of rapid identification of the etiology of acute dyspnea using PoC-LUS on patients’ outcome in the ED [16].

Beside the logistical limitation, there are also risks associated with the transport of unstable patients to the radiology department [17]. Some of the patients presenting in the ED with dyspnea are too unstable to be transported to the radiology department. For these patients PoC-LUS has the advantage of being available at bedside, decreasing the risks associated with transport. Also, there is a negligible cost of each individual PoC-LUS examination.

Acute dyspnea represents one of the most frequent symptoms among patients presenting in the ED [1] and an accurate differentiation of the cause of the dyspnea is challenging [9]. A large epidemiologic study showed that the most common causes of dyspnea in the ED are lower respiratory tract infections, pulmonary edema and exacerbation of the COPD [18]. Chest X-rays and thoracic CT scans are the most used lung imaging techniques in the ED [19]. On one hand, chest X-ray has moderately low sensitivity and specificity in diagnosing COPD exacerbation and pulmonary edema [20]. On the other hand, thoracic CT scan has good sensitivity and specificity, but the time needed to be performed is long [21]. During the last years PoC-LUS has become an important tool in the assessment of the patients presenting with acute dyspnea in the ED. PoC-LUS/ BLUE protocol has been demonstrated to have a very good sensibility and specificity in diagnosing a variety of etiologies as pulmonary edema, pneumonia and COPD exacerbation in patients presented with acute dyspnea in ED [22].

Our study has several limitations. First, being an observational study, the cause effect relationship cannot be drawn. Second, the chest CT scan and PoC-LUS were not performed simultaneously, but the median delay between them was 3 h.

Additionally, it is possible that the results of our study could be influenced by the fact that the emergency physician performing the PoC-LUS was not blinded to the patient’s medical history, clinical evaluation, or ABG results. However, this reflects the reality of clinical practice.

## Conclusions

In conclusion, for the patients presenting in the ED with acute dyspnea, *emergency physician diagnosis* (including medical history, clinical evaluation, ABGSs and *PoC-LUS*) has very good diagnosis performance compared to *attending physician diagnosis* (which also includes laboratory hematological and biochemical tests, CT scans and other specialist assessments as needed). Also, the time needed to complete *emergency physician diagnosis* is much lower than the time needed to complete the *attending physician diagnosis.* Given its high availability in most of the EDs, it’s a useful tool for the assessment of patients presenting with dyspnea.

## Data Availability

The data that support the findings of this study are not publicly available to protect patient privacy, but they are available from the corresponding author upon reasonable request.
